# Abdominal pregnancy - Case presentation

**Published:** 2015

**Authors:** R Bohiltea, V Radoi, C Tufan, IA Horhoianu, C Bohiltea

**Affiliations:** * Obstetrics and Gynecology Department, University Emergency Hospital Bucharest, Romania “Carol Davila” University of Medicine and Pharmacy, Bucharest, Romania; ** Obstetrics and Gynecology Department, University Emergency Hospital, Bucharest, Romania; *** “Carol Davila” University of Medicine and Pharmacy Bucharest, Romania

**Keywords:** abdominal, pregnancy, laparotomy, placenta, bleeding

## Abstract

**Introduction.** Abdominal pregnancy, a rare diagnosis, belongs to the ectopic pregnancy group, the leading cause of pregnancy related exitus. The positive diagnosis is very difficult to establish most often in an acute setting, leading to a staggering percent of feto-maternal morbidity and mortality.

**Case report.** We present the case of 26-weeks-old abdominal pregnancy with partial feto-placental detachment in a patient, after hysteroscopy and in vitro fertilization, which until the acute symptoms that led to emergency laparotomy went unrecognized. The patient recovered completely and satisfactorily after surgery and, due to the high risk of uterine rupture with regard to a second pregnancy, opted for a surrogate mother.

**Conclusion.** Abdominal pregnancy can be regarded as a difficult to establish diagnosis, with a greater chance in case of increased awareness. It is compulsory to be well informed in order not to be surprised by the diagnosis and to apply the correct treatment immediately as the morbidity and mortality rate is elevated.

## Introduction

An ectopic pregnancy can be defined as a pregnancy implantation anywhere else than in the endometrial lining of the uterine cavity [**1**]. The general ectopic pregnancy rate in the population accounts for 2% of first trimester pregnancies with a declining 5% of maternal deaths in developed countries due to improved diagnosis and management; still, it is considered the leading cause of early pregnancy-related exitus [**1**,**2**]. According to [**1**,**2**] the implantation site, classification encompasses the majority of 95% tubal location; the rest of 5% accounting for cervical, cornual, interstitial, ovarian, heterotopic, abdominal or cesarean-scar implantations [**1**-**3**]. For each 1000 ectopic pregnancies, about 9,2 abdominal ones occur [**4**]. The case of a secondary abdominal pregnancy after hysteroscopy and in vitro fertilization is described.

## Case Presentation

**Fig. 1:**
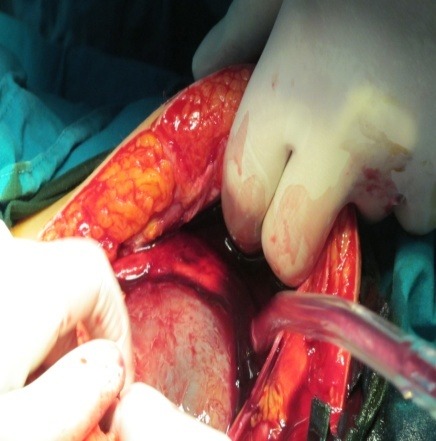
The gestational sac is in direct contact with the anterior wall

**Fig. 2:**
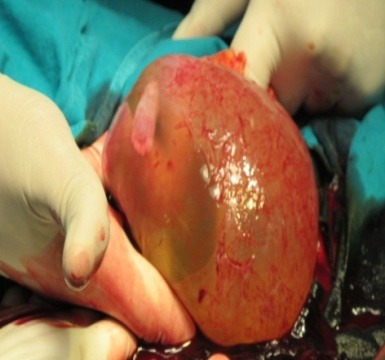
The gestational sac

**Fig. 3:**
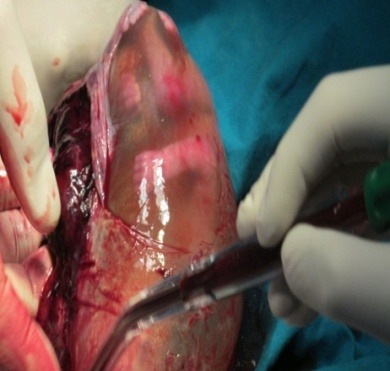
Intraoperatory findings: partially detached fundal uterine placental implantation site

**Fig. 4:**
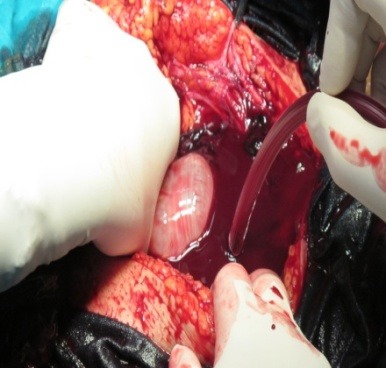
Intraoperatory findings: 600 ml of blood associated with clots and a 23-week-old abdominal pregnancy

**Fig. 5:**
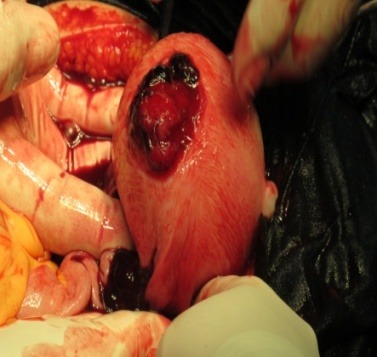
Placental implantation site

A 23-week-old primigravida patient was admitted in the emergency room of the University Emergency Hospital Bucharest for acute pelvic-abdominal pain and lipothymia. Following the immediate stabilization of the vital functions, all the necessary diagnostic steps were taken in order to establish the initial cause. According to her general history, after a normal hysteroscopic evaluation, in vitro fertilization was successfully attained, all prior routine examinations revealing normal results and being uneventful. The emergency ward general and genital examination established a distended painful acute abdomen with a fundal uterine height of approximately 23 weeks without any associated vaginal bleeding or cervical modification. The emergency sonographic evaluation rendered with high suspicion an extrauterine abdominal pregnancy corresponding to the gestational age mentioned - about 23 weeks - without any fetal cardiac activity, an empty uterine cavity and a considerable amount of fluid located in the rectovaginal sac and around the uterus. While the patient’s general state improved, with resumption of lipothymia but persistency of acute abdominal pain with positive signs for peritoneal irritation, the laboratory tests only showed minor anemia (9 g/ dl haemoglobin). Upon establishing the final diagnosis – acute abdomen, suspicion of 23 weeks old abdominal pregnancy with no fetal cardiac activity, transient lipothymia state - an emergency laparotomy was decided. The following intraoperatory findings were discovered: 600 ml of blood associated with clots and a 23-week-old abdominal pregnancy with a partially detached fundal uterine placental implantation site (**Fig. 1**-**5**).

**Fig. 6:**
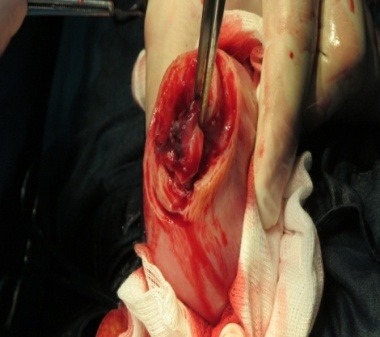
Fundal uterine wedge resection

**Fig. 7:**
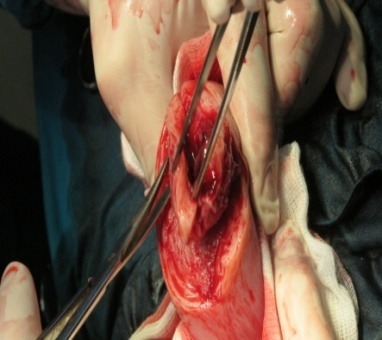
Elimination of the placental villi

A fundal uterine wedge resection successfully eliminated the residual placental villi, which were until the middle third, macroscopically visualized (**Fig. 6**-**7**).

**Fig. 8:**
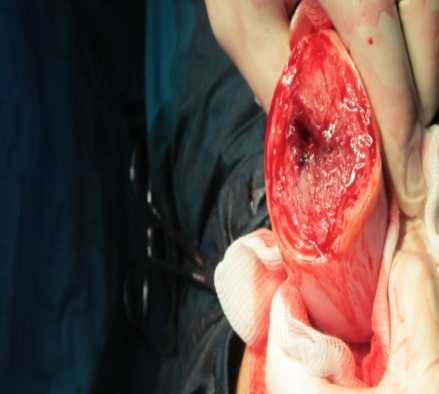
The excised fundal myometrium before applying sutures

**Fig. 9:**
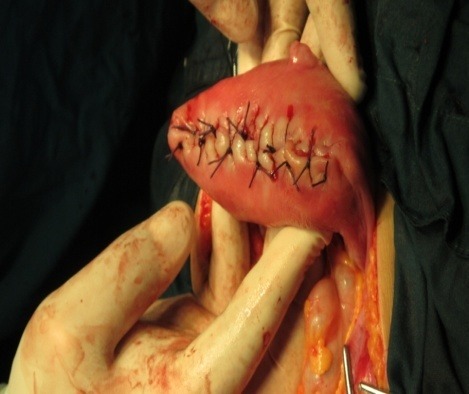
Mattress sutures of the excised fundal myometrium

Mattress sutures were used in order to safely close the excised fundal myometrium (**Fig. 8**-**9**).

No other abdominal findings were observed upon a very thorough examination. The patient recovered without any other complications being released from hospital in 7 days and registering uneventful and satisfactory follow-ups. In order to avoid a premature uterine rupture, a decision regarding a surrogate mother was elected. 

## Discussions

The general rate of abdominal pregnancy is of about 10.9 per 100.00 live births [**4**]. Their number is declining despite a rising trend for ectopic pregnancies; the explanation is lying in the increasing awareness and improved care as the vast majority of abdominal pregnancies are due to ruptured tubal ones [**4**]. The incidence was observed to be higher (with 19%) in non-industrialized countries probably because of the reduced diagnostic options [**5**]. 

Numerous implantation sites have been encountered, among which: uterine, omental, vital organs, large vessels, cul de sac, bowel, appendix, broad ligament, pelvic sidewall, peritoneum, spleen, etc. [**6**-**10**]. In our case, a fundal uterine implantation site occurred with up to the middle third myometrial invasion.

An abdominal pregnancy can go undetected until an advanced gestational age, at which most abdominal pregnancies are discovered, complicating the further management [**7**]. We agree with this assertion, as in our case, the gestational age of 23 weeks was rather advanced and the patients’ survival was attributed to the prompt intervention and favorable uterine implantation site, with only a partial placental detachment. The clinical symptoms of an uncomplicated abdominal pregnancy described in literature are rather unspecific, among which the most frequently encountered: non-labour typically persistent abdominal or suprapubic pain (100%), no delay in menstruation, bloody vaginal discharge, gastrointestinal symptoms like nausea and vomiting (70%), painful fetal movements (40%), general malaise (40%), altered bowel movements [**5**,**7**,**8**,**11**-**14**]. Unfortunately our patient did not experience any of the above-mentioned symptoms, which could have led at least to a more scrupulous and frequent examination with a chance of discovery; the only symptoms experienced being the acute ones due to the bleeding, such as lipothymia and pelvic pain attributed to peritoneal irritation by blood.

The most common physical findings reported in literature are the following: abdominal tenderness (100%), an abnormal fetal lie (70%; breech, oblique or transverse), easily palpating the baby’s parts on clinical examination, and a displaced uterine cervix (40%) [**5**,**12**,**14**,**15**]. Laboratory tests do not have a diagnostic value, among the altered ones, being a positive pregnancy test, elevated human chorionic gonadotropin level or an elevated alpha-fetoprotein with added suspicion [**16**,**17**]. Abdominal tenderness was also positive upon palpation, the other signs being unable to be discovered as the pregnancy in our case was not as advanced despite the fact that the gestational sac was in direct contact with the anterior wall (**Fig. 1**); none of the laboratory tests added suspicion in our case as they presented normal values, exept for the mild anemia.

The final diagnosis, which in our case, is often ultrasonographically obtained with a greater ease at the end of the first trimester or early in the second one, was obtained due to better pelvic organs visualisation [**18**]. Some of the suspicion signs described in literature are listed as it follows: the classic finding represents an empty uterine cavity which can be associated with no sign of ectopic tubal pregnancy, with an abdominal mass representing the fetus without any myometrium surrounding it or with the absence of myometrial tissue between the bladder and the gestational sac and sometimes without any surrounding amniotic fluid; other suggestive ultrasound findings describe the fetal parts being very close to the abdominal wall, an abnormal lie, no amniotic fluid between the placenta and fetus, free intraabdominal fluid or even abnormal doppler velocimetry in the umbilical artery waveform [**6**,**11**,**14**,**16**,**17**,**19**,**20**]. Upon the ultrasonographic evaluation, some of the suspicious signs mentioned above, were found, which led to the correct diagnosis such as the empty uterine cavity and the gestational sac without any myometrium surrounding it located above and anterior to the uterus associated with an increased amount of free fluid – most probably blood. According to [**5**] a positive diagnosis, it can be obtained only if purposely seeking the uterus on a fetal ultrasound examination, with a success rate of 45% of such preoperatory diagnosed cases; also, the insertion of an intrauterine balloon catheter in order to reduce suspicion may help increase the diagnosis [**5**]. Our patient had regular ultrasound follow-ups, all the images revealing a normal pregnancy. We believe this was due to the addition of the 2 separate masses, the empty uterus and the gestational sac with a fundal implantation as one due to the bidimensional image rendered by sonography. Similar facts were presented according to [**14**], a broad ligament pregnancy and empty uterus having been mistaken with a bicornuate uterus pregnancy, (the empty uterine cavity was thought to be the empty horn) [**14**]. Being difficult to diagnose despite the modern echographic techniques listed above, magnetic resonance imaging was used in order to add value, thus lowering the age of identification and locating the fetus and placenta [**7**,**19**,**20**]. Whenever in doubt, diagnostic laparoscopy is also recommended [**19**]. As in our condition, the vast majority of cases unfortunately appear in the emergency ward because of the acute symptoms due to premature rupture of the placental implantation site, so, most frequently, the final diagnosis is obtained upon laparotomy [**7**,**19**]. As showed above, the incidence of the diagnostic error is very high, according to [**12**] of about 60%, signaling the need for an increased awareness and multiple diagnostic procedures in order to lower such a high risk score [**12**].

Because of the severe complications and late diagnosis, the feto-maternal morbidity and mortality rates are also elevated as it follows. The maternal mortality rate varies in literature, from 0,5 to 20%, being about 90 times the maternal mortality rate associated with the normal delivery in the United States [**4**,**5**,**7**,**12**,**14**,**20**,**21**]. According to the literature, maternal morbidity and mortality is associated with severe hemorrhage, bowel obstruction, fistulae or disseminated intravascular coagulation, higher rates being registered whenever the placenta is left in situ as a treatment option [**7**,**12**]. The perinatal mortality classically registers a higher value, of about 40% up until 83-95% [**12**,**14**,**21**]; thanks to recent progresses, the survival rate of fetuses over 30 weeks has grown at about 78-80% [**20**]. Unfortunately, according to multiple sources, between 21 and 90% of the surviving fetuses have serious birth defects due to compression (lack of the amniotic fluid buffer - oligohydramnios) and vascular disruption, some reporting an alarming rate of 50% of the cases, with a survival longer than 1 week; the most frequent findings cited are torticollis, flattening of the head, facial or cranial asymmetry, thorax malformations, limb defects or deficiencies, joint abnormalities or central nervous system malformations [**7**,**14**,**18**,**20**]. In our case presentation, the primary maternal morbidity factor was hemorrhage, which also contributed to the diagnosis with a final favorable resolution, the patient recovering completely and being discharged from hospital in 7 days postoperatively. Unfortunately, as the gestational age was before the achievement of the fetal viability, nothing could be done for the fetus, whose survival was also compromised by the placental detachment (no fetal).

There are many treatment options, all decisions being made in correlation with the intraoperatory findings and gestational age. According to [**7**,**22**], few pregnancies are diagnosed at an early age, at which methotrexate therapy unfortunately proved to be ineffective [**7**,**22**], the sole treatment consisting of the surgical removal by laparotomy, laparoscopy (in the case of poor vascular surfaces) or embolization [**5**,**7**,**14**,**22**]. At the advanced gestational age, at which most abdominal pregnancies are discovered, the management becomes increasingly complicated with a growing feto-maternal morbidity and mortality [**7**]. Laparotomy is the sole treatment option in such cases and, when inspecting the abdominal cavity, the following can be discovered: the placental implantation site, a distorted tube - possibly the primary cause of abdominal pregnancy or mild adhesions especially to the appendix or large bowel [**14**]. According to the literature, various implantation sites have been detected, ranging from the uterus, as in our case, to the posterior surface of the broad ligament, the mesentery or the pouch of Douglas [**5**,**14**,**18**]. According to the intraoperatory findings, which usually define the appearance pattern, abdominal pregnancies can be divided into primary (due to direct intra-abdominal fecundation - 24 cases reported up to 2007 according to [**19**]) and secondary (result of complete tubal abortion, migration of the ovum outwards or missed tubal rupture, being more frequent, with possible intra-laparotomy evidence [**7**,**12**,**14**]). Our case is most probably included in the secondary abdominal pregnancy group, as the most probable cause of outward extrusion was a possible hysteroscopy scar. According to [**14**], secondary pregnancies are more easy to diagnose as there may be early intermittent suprapubic pain, free fluid on the ultrasound examination – which should raise suspicion of a tubal abortion or missed tubal rupture associated with abdominal pregnancy [**14**]. In conclusion, if one pays careful attention to the signs, symptoms and ultrasound findings, a high degree of suspicion is raised, with possible early discovery of such a diagnosis.

After the delivery of the fetus or newborn, the ideal treatment of the placenta, a well debated upon issue, remains to be solved. According to the literature, whenever safely possible (identifying the placental vasculature, and not compromising the blood supply to other organs), the removal of the placenta is a rule, being associated with the best postoperative results and least complications [**5**,**7**,**12**]. The actual procedure consists of an initial ligation of the placental blood supply and afterwards, the removal, because massive life-threatening bleeding can occur due to the absence of a contracting uterus, which generally would occlude the placental bed [**14**]. There are also other treatment options regarding the placenta, like partial removal or leaving the placenta in situ. In case of a partial removal of the placenta, a complete blood supply ligation is needed, if not massive uncontrollable hemorrhage may occur [**22**]. Leaving the placenta in situ with the ligation of the umbilical cord, can be associated with expectant management or other measures, which can accelerate placental trophoblast involution like methotrexate therapy or embolization [**5**,**7**,**12**,**23**]. According to [**18**], after leaving the placenta in situ, the value of beta-human chorionic gonadotropin level can regress to a normal value at 4 moths with a remaining function of about 50 days [**13**,**18**]. Volume regression has also been noted up until 4 weeks, after which no significant reduction was detected [**18**] (from 7.7 x 9.5 x 9.4 cm to 6,3/7,3/6,6 cm). Adjuvant methotrexate therapy is also debated upon, some considering it helpful in accelerating the placental involution, some disapproving its use due to the accumulation of necrotic tissue associated with rapid degradation, with a greater risk of sepsis [**18**]. Leaving the placenta in situ is not advisable as a series of associated complications have been frequently observed, some citing a rate of over 50% (among which over ¾ necessitating blood transfusion) [**18**,**5**]: intestinal obstruction, ileus, fever, infection, peritonitis, prolonged hospital stay, secondary hemorrhage [**7**,**5**,**18**]. In case of intrauterine death, some advocate for a delayed removal in order for the placental vessel atrophy to occur, necessitating a period of up to 3-8 weeks observation [**5**,**18**]. This decision is controversial as there is a high risk of disseminated intravascular coagulation and infection [**5**,**9**,**18**]. When dealing with an abundant hemorrhage besides the general rebalancing embolization associated with manual pressure, sutures or a pedicle graft of the omentum can be helpful, with high precaution so as not to compromise the blood supply to other organs [**5**]. In our case, the placental implantation site was solely on the uterine fundus, which allowed the correct resection within healthy tissue by a fundal wedge resection and the efficient hemostasis, as the uterus is a contracting organ, as showed in the photos below (**Fig. 6**-**9**).

However, there have been some risk factors associated with abdominal pregnancy such as the following: in vitro fertilization as in our case, tubal obstruction, endometriosis, pelvic inflammatory disease, multiparity factors which should aid the positive diagnosis in case of suspicion [**7**,**9**,**15**].

Many pregnancies have achieved full term successfully, together with fetal viability, the last depending on placental vascular supply [**7**]. Many have been delivered successfully [**6**], especially because of the late correct diagnosis; the controversy lies in the treatment option in the case of early abdominal pregnancy detection and in the case of achieving pregnancy viability, whether prolonging or terminating it. Either way, it is compulsory to institute minute follow-ups and correctly inform the patient [**5**,**6**,**14**].

Conclusions – Abdominal pregnancy can be regarded as a difficult diagnosis to establish, with a greater chance in case of increased awareness. It is a very rare condition but its high rate of feto-maternal morbidity and mortality, especially in the emergency ward and associated with advanced pregnancies; make it compulsory to be well informed in order not to be surprised by the diagnosis and to apply the correct treatment immediately.

**Fig. 10:**
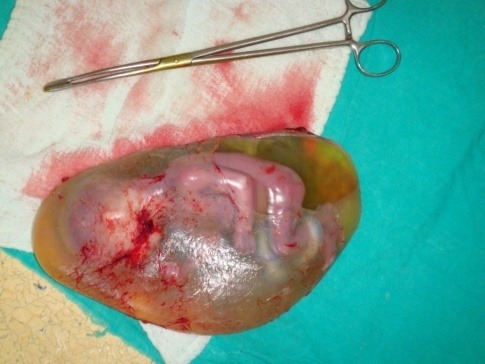
The excised placenta with the fetus

**Fig. 11:**
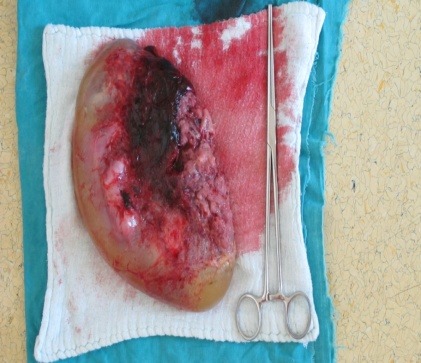
The excised placenta – aspect – the view of the side attached to the implantation site

**Fig. 12:**
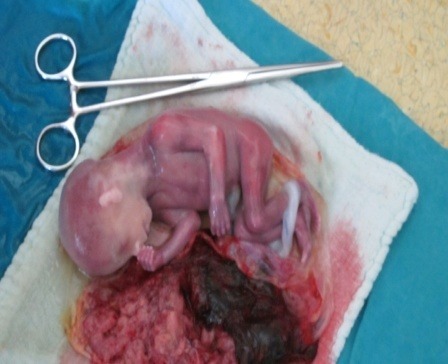
Abdominal pregnancy - the excised placenta and the fetus
